# Discrimination of *Brassica juncea* Varieties Using Visible Near-Infrared (Vis-NIR) Spectroscopy and Chemometrics Methods

**DOI:** 10.3390/ijms232112809

**Published:** 2022-10-24

**Authors:** Soo-In Sohn, Subramani Pandian, Young-Ju Oh, John-Lewis Zinia Zaukuu, Yong-Ho Lee, Eun-Kyoung Shin

**Affiliations:** 1 Department of Agricultural Biotechnology, National Institute of Agricultural Sciences, Rural Development Administration, Jeonju 54874, Korea; 2 Institute for Future Environmental Ecology Co., Ltd., Jeonju 54883, Korea; 3 Department of Food Science and Technology, Kwame Nkrumah University of Science and Technology (KNUST), Kumasi AK-039-5028, Ghana; 4 Institute of Ecological Phytochemistry, Hankyong National University, Anseong 17579, Korea; 5 OJeong Resilience Institute, Korea University, Seoul 02841, Korea

**Keywords:** *Brassica juncea*, visible near-infrared, spectroscopy, deep learning, machine learning, variety discrimination

## Abstract

Brown mustard (*Brassica juncea* (L.) is an important oilseed crop that is mostly used to produce edible oils, industrial oils, modified lipids and biofuels in subtropical nations. Due to its higher level of commercial use, the species has a huge array of varieties/cultivars. The purpose of this study is to evaluate the use of visible near-infrared (Vis-NIR) spectroscopy in combination with multiple chemometric approaches for distinguishing four *B. juncea* varieties in Korea. The spectra from the leaves of four different growth stages of four *B. juncea* varieties were measured in the Vis-NIR range of 325–1075 nm with a stepping of 1.5 nm in reflectance mode. For effective discrimination, the spectral data were preprocessed using three distinct approaches, and eight different chemometric analyses were utilized. After the detection of outliers, the samples were split into two groups, one serving as a calibration set and the other as a validation set. When numerous preprocessing and chemometric approaches were applied for discriminating, the combination of standard normal variate and deep learning had the highest classification accuracy in all the growth stages achieved up to 100%. Similarly, few other chemometrics also yielded 100% classification accuracy, namely, support vector machine, generalized linear model, and the random forest. Of all the chemometric preprocessing methods, Savitzky–Golay filter smoothing provided the best and most convincing discrimination. The findings imply that chemometric methods combined with handheld Vis-NIR spectroscopy can be utilized as an efficient tool for differentiating *B. juncea* varieties in the field in all the growth stages.

## 1. Introduction

Brassica is a genus of plants in the Brassicaceae family. The Brassicaceae family contains approximately 3709 species and 338 genera and is utilized as a source of oil, vegetables, mustard sauces, and fodder [1,2]. *B. napus*, *B. rapa*, and *B. juncea* are members of this seed family that have a strong industrial interest in the oil extraction industries [3]. In tropical and subtropical nations, particularly south-east Asia such as India, China, Bangladesh, and Pakistan, and parts of Canada, Russia, China, and Australia, *Brassica juncea* (L.) Czern & Coss (Indian mustard) is a significant oilseed crop [2]. It is a natural amphidiploid (AABB, 2n = 36) of *Brassica rapa* (AA, 2n = 20) and *Brassica nigra* (BB, 2n = 16) that is farmed for its edible oil globally [4]. In addition to being used in cooking, Indian mustard has a wide range of uses in the food and chemical industries, as well as being utilized as a biofertilizer. Mustard seedmeal is an excellent feed for poultry animals, and India has become the world’s largest exporter [5]. Mustard oil has a rich repertoire of antioxidants and high erucic acid, as well as excellent lubricating and combustion qualities, and is thus widely used and desired in biodiesel production, the automobile industry, and the paint industry [6].

Recently, the “Industry 4.0” era has necessitated the development of non-destructive and environmentally friendly procedures for the simple, rapid, and accurate assessment of varieties/species based on their composition and oil content. Visible near-infrared (Vis-NIR) spectroscopy is a vibrational spectroscopy technique that relies on the absorption of electromagnetic radiation in the visible and NIR range (350–2500 nm) to provide information about molecular vibrations of chemical bonds involving primary structural components of molecules [7]. This technique has been reported to discriminate plant species/varieties in various crops, such as tea [8], apple [9], peach [10], Amaranthus species [11], etc. In addition, it is used to predict oil content in soybean [12], sugar beet seed [13], sesame seed [14], and *B. napus* seed [15]. New sensors, such as portable NIR spectrometers, are currently being evaluated in a variety of agricultural products [16,17]. Due to their small size, these sensors are comfortable and portable, allowing them to monitor the industry at various phases of the supply chain, from harvesting to processing. Multivariate analysis techniques are frequently employed to extract crucial information from NIR data due to the large amount of data generated [18]. Principal component analysis (PCA) is used to obtain a rapid overview of the spectra, whilst multivariate calibration methods such as Discriminant Analysis (PLS-DA), deep learning and Partial Least Squares Regression (PLSR) allow for the classification and prediction of desired parameters in samples, respectively [19,20]. In this study, the specific objectives were to (1) evaluate the capacity of portable Vis-NIR spectroscopy to discriminate plant varieties and (2) compare the eight chemometric methods and their combinations with various preprocessing techniques for effective discriminating of four different *B. juncea* varieties.

## 2. Results and Discussion

### 2.1. Diffuse Reflectance Spectroscopic Analysis and Preprocessing

**Figure 1:**
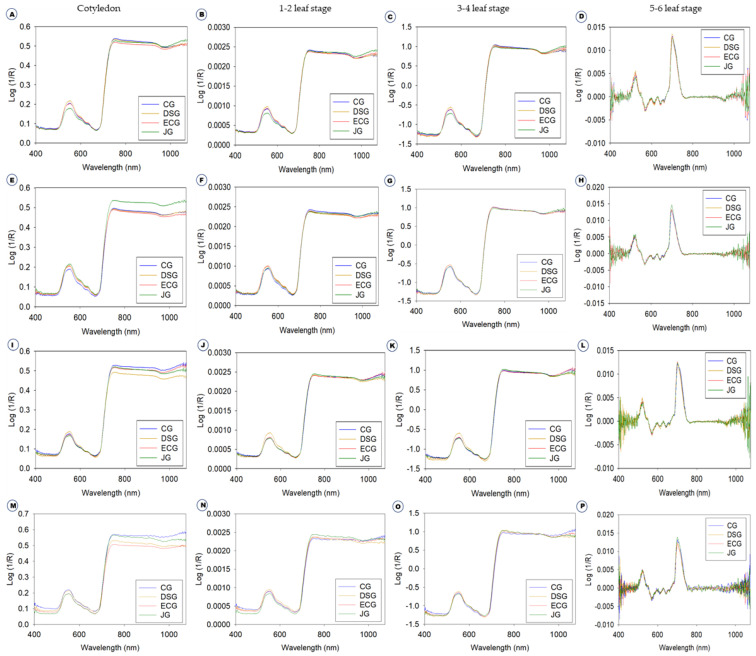
Average raw and preprocessed spectra of four growth stages of four *B. juncea* varieties. Average raw (**A**,**E**,**I**,**M**) and preprocessed with different preprocessing methods, namely, normalization (**B**,**F**,**J**,**N**), standard normal variate (**C**,**G**,**K**,**O**), and Savitzky–Golay (**D**,**H**,**L**,**P**).

**Figure 2:**
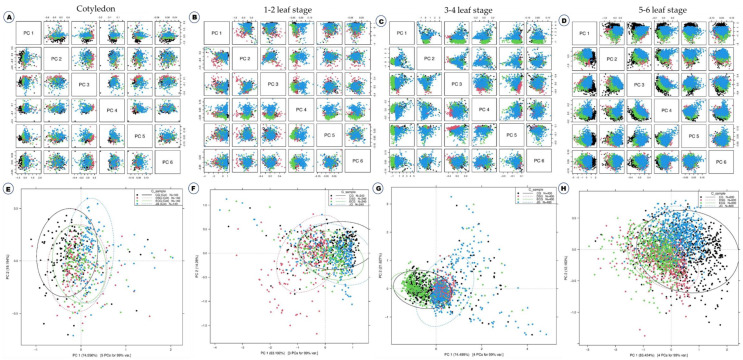
Principal component analyses based on the Vis-NIR spectra of four different growth stages of four *Brassica juncea* varieties. Raw spectra have been used. (**A**–**D**) Paired blot; (**E**–**H**) axes are the first and second principal components.

Figure 1 shows the average Vis-NIR spectra obtained from the four different growth stages of four *B. juncea* varieties, namely, cotyledon stage (Figure 1A,E,I,M), 1–2 leaf stage (Figure 1B,F,J,N), 3–4 leaf stage (Figure 1C,G,K,O) and 5–6 leaf stages (Figure 1D,H,L,P). This includes raw spectra and three different preprocessing methods. There are numerous crossovers and overlapping across the four varieties in all the growth stages (Figure 1A–P); in other words, the spectra of each variety are quite similar to those of other varieties. Consequently, the discrimination of varieties directly based on absorbance spectra is difficult. Therefore, it was necessary to use machine learning methods for the effective discrimination of four varieties. From 400 to 500 nm, the spectral curve was flat, and between 550 and 650 nm there was a small peak and again down to their normal position. This demonstrates that the leaves actively absorb blue (400–500 nm) and red (680 nm) light while reflecting green light (550 nm) in the visible range [8] which is responsible for chlorophylls and carotenoids [21,22]. From 650 to 750 nm, there was a sharp increase in the peak that remained higher absorbance value; later, there are no variations in remaining wavelength until 1200 nm. These results concurred with our previous research on the discrimination of *B. napus* and *B. juncea* using Vis-NIR spectroscopy [23]. The spectra were preprocessed to reduce systemic noise and emphasize differences between samples. Using a number of preprocessing methods simultaneously will help us obtain a greater degree of classification accuracy and will allow us to select the best preprocessing approach for each sample [23,24]. It is difficult to discriminate the plant varieties only with the spectra shown in Figure 1. For effective discrimination, Vis-NIR spectroscopy was combined with several models and machine learning methods such as discriminant analysis and principal component analysis (PCA) is important [20,25]. To investigate the qualitative differences between the four *B. juncea* varieties, PCA was performed using raw spectra (Figure 2). PCA is a powerful data mining technique for data visualization. The principle of PCA is to determine the linear combinations of the initial variables that contribute to the differences between samples [26]. These combinations are referred to as principal components (PCs). As shown in Figure 2A–D, all of the different PCs showed the same slight pattern of separation for the different samples in the PCA paired plot from PC1 to PC6, but PC1 vs. PC2 (Figure 2E–H), showed the most visual differences in different growth stages, respectively. Therefore, outlier detection was performed using these two PCs before initiating preprocessing for the machine learning methods. Generally, the computerized iterations allow PC1 to have the maximum information and PC2 to carry the maximum share of residual information [26].

### 2.2. Chemometric Analysis for Discrimination of Four B. juncea Varieties

The potential of visible-NIR spectroscopy to discriminate or identify plant varieties are based on leaf spectral properties related to biochemical composition and structure, which are influenced by a variety of factors such as plant species, development or microclimate position of the leaf on the plant, etc. [21,27]. To determine the most accurate method for distinguishing four *B. juncea* varieties, the classification accuracy of various chemometric methods combined with different preprocessing methods was assessed. Table 1 shows a summary of the classification accuracy for the various methods in different growth stages of four *B. juncea* varieties. The classification accuracies ranged from 45.0% to 100.0%. Using chemometric approaches, both raw and preprocessed spectra displayed efficient discrimination with different classification accuracies.

In most chemometric analyses, however, preprocessed spectra were found to have a higher classification accuracy than raw spectra. In some cases, the use of raw spectra yielded much less classification accuracy with the use of Decision tree (45.0%), Random Forest (45.4%) and Naïve Bayes (48.0%). The maximum classification accuracy (100%) was witnessed with the several preprocessing methods in combination with machine learning methods (Table 1). Especially during the 5–6 leaf stage of plants the classification accuracy is highest, sometimes. even without preprocessing.

In assessing the effectiveness of classification methods, deep learning, SVM and linear discriminant analyses were found to have higher level of classification accuracy. Naïve Bayes and decision tree had the lowest accuracies. Notably, classifications using only raw spectra still yield average accuracies above 70% at the Cotyledon stage, 1–2 leaf stage, 3–4 leaf stage and 5–6 leaf stage when using Generalized Linear Model, Fast Large Margin, Deep Learning, Decision Tree, SVM and linear discriminant analysis. Without preprocessing the data, the SVM model had a high accuracy of 100% at 5–6 leaf stage. The SVM is particularly well suited to high-dimensional data, because the value of each attribute is arbitrary [28].

In assessing the effectiveness of preprocessing on classification, Standard Normal Variate produced the best classification accuracies in combination with all the other classification methods in most cases. Normalization and Savitzky–Golay (derivative) produced acceptable accuracies (Table 1) depending on the classification method that they were used in combination with. Previously, various studies used a variety of preprocessing and chemometric approaches to differentiate plant species. Yee et al. [29] employed NIR spectra in conjunction with LDA to discriminate potato tuber varieties, with a classification accuracy of 93%. Chen et al. [30] used SVM to differentiate three tea varietals. Similarly, Vis-NIR spectroscopy paired with artificial neural networks (ANN) successfully distinguished tea plants with a 77.3% accuracy [8]. For on-site tomato variety discrimination, Xu et al. [21] used PCA, linear discriminant analysis (LDA), and discriminant partial least squares (DPLS) regression approaches.

**Figure 3:**
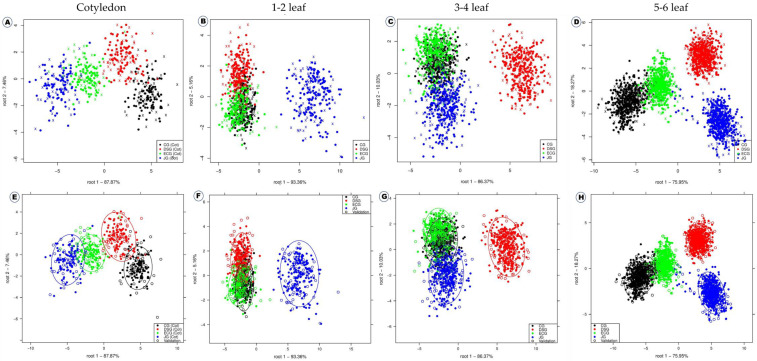
Linear discriminant analysis for the effective discrimination of four growth stages of four *B. juncea* varieties without confidence circles (**A**–**D**) and with confidence circles (**E**–**H**).

Overall, the combination of SNV and deep learning was found to be more effective in the discrimination of four *B. juncea* varieties in all the growth stages in our study. The SNV (100%) was the most effective preprocessing approach for usage with several chemometric methodologies. The linear discriminant analysis plot for the discrimination of four *B. juncea* varieties is shown in Figure 3. The distribution of spectral points and their compactness varies according to the growth stages. The 5–6 leaf stage of *B. juncea* varieties was found to be a promising stage for the variety discrimination. The variety “Jukgot” was completely separated from the clusters of other varieties, while clusters of other varieties were closely placed. This implies that the other three varieties share higher levels of biological composition, but “Jukgot” shares much less with other varieties. Similarly, LDA was utilized to discriminate between numerous plant varieties, including sprouting mung bean [31] and melon cultivars [32].

### 2.3. Selection of Significant Preprocessing and Chemometric Methods for Discrimination

The effectiveness of preprocessing and machine learning methods was statistically evaluated (Table 2). The mean percentage of classification accuracy of each chemometric method paired with various preprocessing procedures revealed significant modeling for the discrimination of four *B. juncea* varieties (Table 2). The statistical analysis using analysis of variance (ANOVA) demonstrated that the sum of square and mean sum of square values of the various preprocessing and machine learning techniques used had statistical significance at *p ≤* 0.0001 (Table 3). However, when a combination of preprocessing and multiple machine learning approaches was used, there was no significance with *p ≤* 0.0001. (*p* value of 0.0389). The confusion matrix illustrates the degree of error in the identification of the assessed plants, suggesting that SNV combined with deep learning was the most accurate classification method (Tables S1–S4). Similar results were witnessed by the use of Vis-NIR spectroscopy in the discrimination of *Amaranthus* sp. [11] and hybrids between *B. napus* and *B. juncea* [23].

## 3. Materials and Methods

### 3.1. Plant Materials

**Figure 4:**
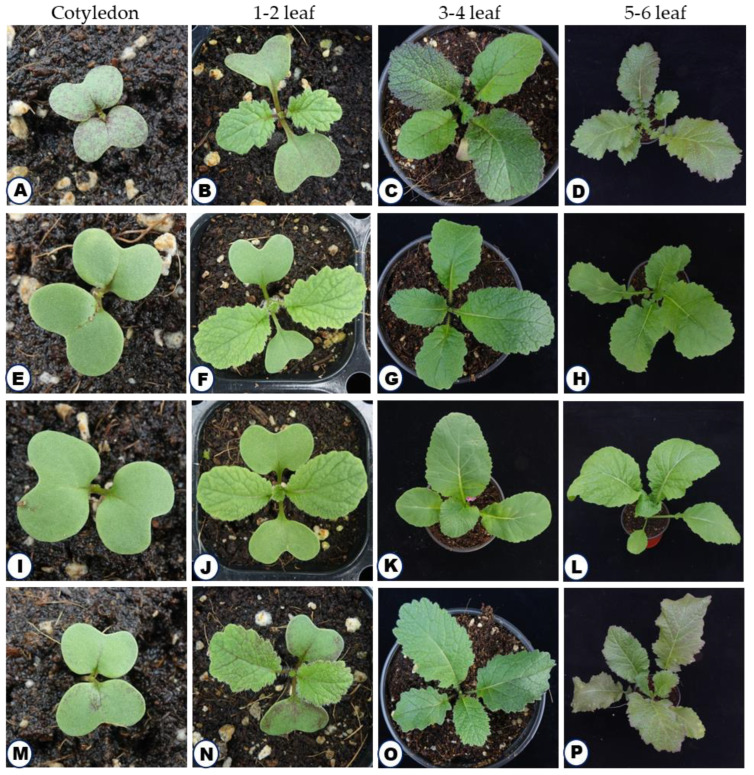
Representative figures of four different growth stages of four *Brassica juncea* varieties used in the study. (**A**–**D**), Jukgot; (**E**–**H**), Chungot; (**I**–**L**), Dolsangot; (**M**–**P**), Earlchungot. Growth stages cotyledon, 1–2 leaf stage, 3–4 leaf stage, 5–6 leaf stage, respectively.

Four *B. juncea* L. varieties of the Korean peninsula with the following local names: ‘Jukgot’ ‘Chungot’ ‘Dolsangot’ and ‘Earlchungot’ were selected for the discrimination analysis using Vis-NIR spectroscopy. All the four varieties were purchased from the Asia Seed Co., Ltd. Seoul, Republic of Korea. All the varieties were grown in the soil pot at the greenhouse of the National Institute of Agricultural Sciences, Jeonju, Republic of Korea, during May–July 2021. The discrimination analysis was performed with different growth stages of the *B. juncea* plants, namely, cotyledon stage, 1–2 leaf stage, 3–4 leaf stage and 5–6 leaf stages (Figure 4).

### 3.2. Vis-NIR Spectral Data Collection

Vis-NIR diffuse reflectance spectra of intact leaves of four *B. juncea* varieties were acquired using a handheld integrated portable spectrum analyzer (FieldSpec HandHeld 2, ASD Inc., Longmont, CO, USA) in the range of 325–1075 nm with a stepping of 1.5 nm in reflectance mode (log/R). The spectra were taken on the fully inflated leaves’ adaxial surface, which may easily capture light. In each group, the spectra were acquired from three distinct sections of the leaf blade. Three spectra were obtained from various parts of the leaf blade of hundred plants in each group. A total of 300 (3 × 100 = 300) spectra were collected from each group and used for further analysis. The leaf of the cotyledon stage is very small the spectral collection is difficult; therefore, we performed collection in a single section (1 × 100 = 100). To remove unnecessary noise, the Vis-NIR device’s optical window was placed directly on the leaf’s face during each spectrum capture, assuring that the sensor window was entirely covered.

### 3.3. Preprocessing, Modelling Methods and Statistical Analysis

Background signals arose in the raw spectra of samples due to system settings and external noise. As a result, numerous preprocessing procedures, such as normalization (area), standard normal variate (SNV), and derivatives (Savitzky–Golay with first differentiation), were used to reduce spectral noise and improve the accuracy of modeling approaches [20,23]. The efficiency of preprocessing methods was evaluated in comparison to raw spectra. The preparation computations were carried out using the Unscrambler X program, version 10.5.1. (CAMO ASA, Oslo, Norway). Several machine learning algorithms were used and compared for effective spectral data visualization and discrimination. The modeling was performed with RapidMiner studios Version 9.0.002 (RapidMiner, Inc., Boston, MA, USA). Deep learning, decision trees, support vector machines (SVM), random forests, generalized linear model, rapid large margin, Naïve Bayes, and linear discriminant analysis were used in this study to find the best modeling technique with the highest classification accuracy [20,23]. The Aquap2 package created by Pollner and Kovacs [33] was also utilized in R-studio to apply the various preprocessing approaches and perform linear discriminant analysis. The spectral data points were the inputs for each approach, and the classes were the identifying labels for four *B. juncea* varieties. Cross validation was used to test the models’ predictability across several sample types. For this, the data were separated into two sets: a training set and a validation set. The training set contained two-thirds of the data, with the remainder serving as the validation set. The data were split three times to ensure that each sample was evaluated at least once in the calibration and validation set. Using one-way analysis of variance, the influence of (1) the scatter correction method, (2) the eight machine learning methods, and (3) the interaction between preprocessing and machine learning methods was identified (ANOVA). Tukey’s range test was employed as a mean comparison procedure with a significance level of *p ≤* 0.05.

## 4. Conclusions

In conclusion, using Vis-NIR spectroscopy in combination with several machine learning approaches, a simple and rapid discrimination method for *B. juncea* varieties was established. Among the various preprocessing and machine learning approaches used, the combination of standard normal variate and deep learning proved to be the most accurate, with a 100% classification accuracy of *juncea* varieties at the 5–6 leaf stage and accuracies higher than 89%, irrespective of the growth stage. However, when compared with the standard normal variate, the Savitzky–Golay smoothing performed well with other chemometrics, indicating that it has better discrimination potential when utilizing several chemometric approaches. Especially, the discrimination accuracy is higher in the 5–6 leaf stage compared with other stages. Furthermore, it is confirmed that this nondestructive technique, which combines handheld Vis-NIR spectroscopy with chemometric techniques, can be utilized to distinguish between different plant varieties in the field for rapid identification. It is also advised that a database containing large-scale germplasm collections of *B. juncea* and/or other plant varieties be created for effective global use of the technology.

## Figures and Tables

**Table 1:** Average classification accuracy of the combinations of preprocessing and machine learning methods for reflectance spectra from four different growth stages of eight *B. juncea* varieties.

S. No	Model	Preprocessing	Average Accuracy (%)			
			Cotyledon Stage	1–2 Leaf Stage	3–4 Leaf Stage	5–6 Leaf Stage
1	Naïve Bayes	Raw spectra	56.2	55.5	59.1	55.2
		Normalization (Area)	61.4	48.0	58.2	58.6
		Standard Normal Variate	79.7	62.4	60.8	73.2
		Savitzky–Golay (Derivative)	60.0	73.5	77.1	99.8
2	Generalized Linear Model	Raw spectra	70.0	70.9	71.2	80.7
		Normalization (Area)	69.0	78.3	83.7	86.2
		Standard Normal Variate	82.8	81.6	85.1	98
		Savitzky–Golay (Derivative)	76.2	74.1	79.1	100
3	Fast Large Margin	Raw spectra	82.8	85.4	87.7	99.8
		Normalization (Area)	62.8	52.0	68.7	65.8
		Standard Normal Variate	83.1	87.7	91.1	100
		Savitzky–Golay (Derivative)	63.8	73.5	86.0	99.9
4	Deep Learning	Raw spectra	80.3	84.3	87.0	98.2
		Normalization (Area)	82.8	86.4	87.8	99.9
		Standard Normal Variate	89.0	89.1	92.0	100
		Savitzky–Golay (Derivative)	71.7	77.6	88.1	100
5	Decision Tree	Raw spectra	60.3	57.6	54.5	63.5
		Normalization (Area)	65.2	50.5	45.0	67.1
		Standard Normal Variate	71.7	65.4	54.2	82.0
		Savitzky–Golay (Derivative)	45.2	72.5	76.5	51.2
6	Random Forest	Raw spectra	61.0	58.5	59.9	59.2
		Normalization (Area)	72.8	45.4	65.4	72.3
		Standard Normal Variate	85.9	71.3	81.3	86.8
		Savitzky–Golay (Derivative)	65.9	73.5	77.2	100
7	Support Vector Machine	Raw spectra	85.9	86.1	88.6	100
		Normalization (Area)	80.0	78.1	80.3	73.2
		Standard Normal Variate	88.6	89.2	91.3	100
		Savitzky–Golay (Derivative)	66.6	76.5	86.8	100
8	Linear Discriminant Analysis	Raw spectra	83.4	79.9	84.9	99.5
		Normalization (Area)	86.4	80.6	81.7	99.6
		Standard Normal Variate	87.3	80.6	84.9	99.8
		Savitzky–Golay (Derivative)	92.5	91.7	86.9	99.6

**Table 2:** Model precisions of different preprocessing and models for the discrimination of four *B. juncea* varieties.

	Raw	Normalize	Savitzky–Golay	SNV	*p*-Value
Deep Learning	85.45 ± 0.04 a	86.55 ± 0.04 a	78.55 ± 0.06 a	90.11 ± 0.03 ab	NS
Decision Tree	47.27 ± 0.08 bc	46.37 ± 0.08 c	31.98 ± 0.09 b	53.17 ± 0.08 c	NS
Fast Large Margin	86.36 ± 0.04 Aa	55.52 ± 0.07 Bc	67.55 ± 0.09 B a	87.01 ± 0.03 Aab	**
Generalized Linear Model	62.80 ± 0.07 b	76.51 ± 0.05 a	71.98 ± 0.07 a	82.68 ± 0.04 ab	NS
Naïve Bayes	41.70 ± 0.07 c	51.56 ± 0.06 c	56.05 ± 0.11 a	57.20 ± 0.08 c	NS
Random Forest	51.73 ± 0.06 bc	58.03 ± 0.06 bc	58.96 ± 0.10 a	74.18 ± 0.06 b	NS
Support Vector Machine	88.79 ± 0.03 Aa	74.15 ± 0.04B ab	75.63 ± 0.06 Ba	91.33 ± 0.03 Aa	**
*p*-value	***	***	**	***	

NS, not significant, **, *p <* 0.01, ***, *p <* 0.001. Means with different alphabetical small and capital letters show the significance of the value in the order of column (machine learning) and row (preprocessing), respectively. Same letters are not significantly different at *p* ≤ 0.05 based on Tukey’s range test.

**Table 3:** Analysis of variance of percentage of correctly classified four *B. juncea* varieties from four different preprocessing methods and eight different classification models using reflectance spectra.

Source	DF	SS	MS	*f* Value	*p*-Value
Stage	3	4.800979	1.600326	28.26	<0.0001
Pretreatment	3	1.288898	0.429633	7.59	<0.0001
Model	6	9.174862	1.529144	27	<0.0001
Stage × Pretreatment	9	0.678759	0.075418	1.33	0.2192
Stage × Model	18	0.932161	0.051787	0.91	0.5614
Pretreatment × Model	18	1.725652	0.09587	1.69	0.0389
Stage × Pretreat × Model	54	2.605497	0.04825	0.85	0.7601
Error	336	19.03025	0.056638		
Total	447	40.23706			

DF: degree of freedom. SS: sum of squares. MS: mean sum of squares.

## Data Availability

Not applicable.

## Supplementary Materials

The following supporting information can be downloaded at: https://www.mdpi.com/article/10.3390/ijms232112809/s1.
