# Supplementary material for: Discrimination of Brassica juncea Varieties Using Visible Near-Infrared (Vis-NIR) Spectroscopy and Chemometrics Methods
Source: Int J Mol Sci. 2022 Oct 24;23(21):12809. doi: 10.3390/ijms232112809 (PMC9654150; doi:10.3390/ijms232112809)
Supplement: Supplementary file 1 [file ijms-23-12809-s001.zip › ijms-1969765-supplementary.pdf]

Table S1. Confusion matrix from the execution with the best accuracy in cotyledon stage of four *B. juncea* varieties (**Deep learning/SNV).**

	Chungot	Dolsangot	Earlchungot	Jukgot	class precision
Chungot	36	2	1	0	92.31%
Dolsangot	5	57	2	2	86.36%
Earlchungot	0	13	41	5	69.49%
Jukgot	0	0	2	124	98.41%
class recall	87.80%	79.17%	89.13%	94.66%	

Table S2. Confusion matrix from the execution with the best accuracy in 1-2 leaf stage of four *B. juncea* varieties (**SVM/SNV).**

	Chungot	Dolsangot	Earlchungot	Jukgot	class precision
Chungot	5	1	5	0	45.45%
Dolsangot	55	198	55	0	64.29%
Earlchungot	10	7	20	0	54.05%
Jukgot	0	0	0	211	100.00%
class recall	7.14%	96.12%	25.00%	100.00%	

Table S3. Confusion matrix from the execution with the best accuracy in 3-4 leaf stage of four *B. juncea* varieties (Deep learning/SNV).

	Chungot	Dolsangot	Earlchungot	Jukgot	class precision
Chungot	165	0	27	43	70.21%
Dolsangot	0	453	0	0	100.00%
Earlchungot	4	0	88	6	89.80%
Jukgot	21	0	9	562	94.93%
class recall	86.84%	100.00%	70.97%	91.98%	

Table S4. Confusion matrix from the execution with the best accuracy in 5-6 leaf stage of four *B. juncea* varieties (Deep learning/SNV).

	Chungot	Dolsangot	Earlchungot	Jukgot	class precision
Chungot	202	0	0	0	100.00%
Dolsangot	0	236	0	0	100.00%
Earlchungot	0	0	207	0	100.00%
Jukgot	0	0	0	210	100.00%
class recall	100.00%	100.00%	100.00%	100.00%	
